# A systems biology analysis of adrenergically stimulated adiponectin exocytosis in white adipocytes

**DOI:** 10.1016/j.jbc.2021.101221

**Published:** 2021-09-29

**Authors:** William Lövfors, Christian Simonsson, Ali M. Komai, Elin Nyman, Charlotta S. Olofsson, Gunnar Cedersund

**Affiliations:** 1 Department of Biomedical Engineering, Linköping University, Linköping, Sweden; 2 Department of Mathematics, Linköping University, Linköping, Sweden; 3 Department of Physiology/Metabolic Physiology, Institute of Neuroscience and Physiology, University of Gothenburg, Gothenburg, Sweden; 4 Center for Medical Image Science and Visualization, Linköping University, Linköping, Sweden

**Keywords:** adiponectin, exocytosis, mathematical modeling, adrenergic signaling, white adipocytes, diabetes, systems biology, β_3_AR, adrenergic β_3_ receptor, EC, extracellular, Epac1, exchange protein directly activated by cAMP, isoform 1, EPI, epinephrine, ODE, ordinary differential equation, PM, plasma membrane, VAMP, vesicle-associated membrane protein

## Abstract

Circulating levels of the adipocyte hormone adiponectin are typically reduced in obesity, and this deficiency has been linked to metabolic diseases. It is thus important to understand the mechanisms controlling adiponectin exocytosis. This understanding is hindered by the high complexity of both the available data and the underlying signaling network. To deal with this complexity, we have previously investigated how different intracellular concentrations of Ca^2+^, cAMP, and ATP affect adiponectin exocytosis, using both patch-clamp recordings and systems biology mathematical modeling. Recent work has shown that adiponectin exocytosis is physiologically triggered *via* signaling pathways involving adrenergic β_3_ receptors (β_3_ARs). Therefore, we developed a mathematical model that also includes adiponectin exocytosis stimulated by extracellular epinephrine or the β_3_AR agonist CL 316243. Our new model is consistent with all previous patch-clamp data as well as new data (collected from stimulations with a combination of the intracellular mediators and extracellular adrenergic stimuli) and can predict independent validation data. We used this model to perform new *in silico* experiments where corresponding wet lab experiments would be difficult to perform. We simulated adiponectin exocytosis in single cells in response to the reduction of β_3_ARs that is observed in adipocytes from animals with obesity-induced diabetes. Finally, we used our model to investigate intracellular dynamics and to predict both cAMP levels and adiponectin release by scaling the model from single-cell to a population of cells—predictions corroborated by experimental data. Our work brings us one step closer to understanding the intricate regulation of adiponectin exocytosis.

The white adipocyte hormone adiponectin is a major regulator of glucose and lipid homeostasis, with direct effects on the liver, muscle, the vasculature, and the pancreas (1, 2). Adiponectin levels are decreased in obese/type 2 diabetic individuals, and preserved adiponectin levels are associated with a reduced risk of developing metabolic disease (3, 4).

Although much is known regarding the important pathophysiological roles of adiponectin, the mechanisms regulating its secretion from white adipocytes are less well studied. Our previous experimental work has defined a secretory pathway where adiponectin-containing vesicles are released in response to an elevation of intracellular cAMP and activation of Epac1 (*E*xchange *P*rotein directly *A*ctivated by *c*AMP, isoform 1). In addition, both Ca^2+^ and ATP have important roles for augmentation of adiponectin release as well as for maintenance of adiponectin secretion over extended time periods (5, 6). Thus, the same mediators that are important for secretion of conventional hormones are involved in the regulation of adiponectin exocytosis (although peptide/protein hormone secretion is typically stimulated by an elevation of cytosolic Ca^2+^) (7). In later work, we showed that adiponectin secretion is physiologically stimulated *via* adrenergic signaling, chiefly involving beta3 adrenergic receptors (β_3_ARs). The same study demonstrates that adiponectin release is blunted in adipocytes from obese and diabetic mice, due to lower abundance of β_3_ARs in a state of *catecholamine resistance* (8). Clearly, the pathophysiological control of adiponectin secretion involves a highly complex signaling network of not yet fully understood interactions.

To deal with this high complexity, we have employed a systems biology approach combining experiments with mathematical modeling. We have previously developed a mathematical model for single-cell adiponectin exocytosis in 3T3-L1 adipocytes (9). In this previous work, changes in plasma membrane capacitance were measured using the patch-clamp technique. As vesicles fuse with the plasma membrane upon exocytosis, the membrane area is enlarged, and this can be measured as an increase in capacitance (the separation of charges over the plasma membrane) (10). Capacitance recordings can thus provide detailed information about the exocytosis process, at high time resolution and in live single cells, including adipocytes (11). In our previous work, capacitance measurements were utilized to model how vesicle exo- and endocytosis are affected by different combinations of intracellular Ca^2+^, cAMP, and ATP concentrations (9). The concentrations of intracellular mediators were altered by inclusion of different concentrations in the patch pipette attached to the cell. During the electrophysiological recordings, the pipette is in direct contact with the cell interior, allowing exchange between the pipette filling solution and the cell cytosol. We routinely measure adipocyte exocytosis in cultured adipocytes by this approach, and our studies have demonstrated that the cAMP-triggered and Ca^2+^-augmented capacitance increase in 3T3-L1 adipocytes largely represents secretion of adiponectin (5, 6, 8). Our model in (9) allowed us to extract detailed information contained in patch-clamp experiments and thus estimate the relative differences in adiponectin exo-and endocytosis rates. We could also correctly predict adiponectin secretion stimulated by cAMP in the presence or absence of Ca^2+^ or ATP. However, effects of extracellular cues, such as adrenergic stimulation, were not included in our previous model. This is an important shortcoming, since catecholamine stimulation constitutes a central stimulus-secretion pathway for adiponectin in normal physiological conditions.

In the current study, we have extended the model in Brännmark 2017 (9) to also include external, receptor-mediated, adrenergic stimulation. The resulting model can describe all previous capacitance data as well as new data of stimulation with epinephrine or the β_3_AR agonist CL 316243 (CL). The model can explain data used for training, as well as accurately predict independent validation data. Simulations with the new and improved model allow us to better understand the mechanisms involved in the adrenergic control of adiponectin exocytosis.

## Results

### Development of a new model including both pipette and external stimuli

**Figure 1:**
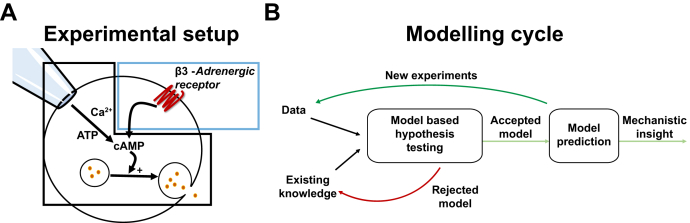
**Overview of the experimental approach and the modeling cycle.***A*, the main variables of our previously published model of adiponectin exocytosis are contained within the *black box*, and the extension with adrenergic stimulus within the *blue box*. *B*, a schematic overview of the model development cycle. Experimental data and existing knowledge are used to construct and test a hypothesis using mathematical simulations. If the model is rejected, the hypothesis must be revised. If it is not rejected, the model is used to predict new experiments, which are later tested experimentally. A model that can accurately predict new experiments can be used to generate new insights into the detailed regulation of adiponectin vesicle release.

**Figure 2:**
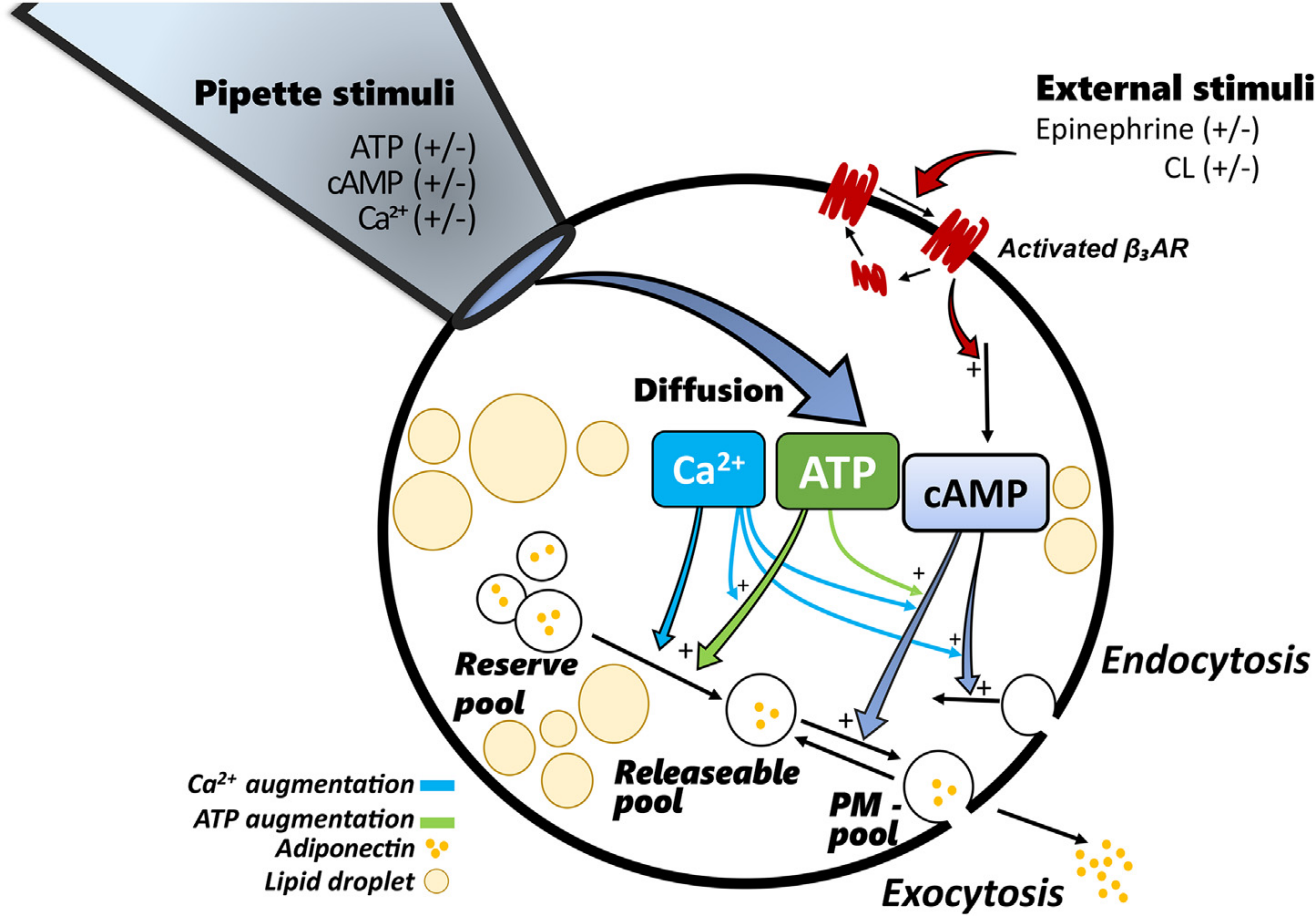
**Detailed overview of our extended model.** Stimulus in the model occurs either *via* the pipette solution (ATP, cAMP, and Ca^2+^) or externally (epinephrine or CL). The pipette stimulus is allowed to diffuse between the pipette and the inside of the cell, while the external stimulus leads to activation of the receptor, which in turn leads to an increase in cytosolic cAMP. The transition of vesicles from the reserve pool to the releasable pool is stimulated by Ca^2+^, either alone or augmented with ATP. The fusion of adiponectin vesicles residing in the releasable pool is triggered by cAMP and augmented by both Ca^2+^and ATP. Vesicles in the PM pool can either go back to the releasable pool or be exocytosed, independent of stimuli. Endocytosis is stimulated by Ca^2+^ but must be preceded by an elevation of cAMP (to trigger exocytosis). All equations and simulation files are available as described under the Data availability section.

By enlarging the pool of available data and evolving the mechanistic hypothesis, we expanded the model in (9) to include external receptor-mediated adrenergic stimulation (Fig. 1*A*). More specifically, we tested if the model could be improved to produce an acceptable agreement to the new expanded dataset, by doing several iterations of the lower branch in Figure 1*B*. First, the original model was extended to include a more accurate representation of the pipette, where diffusion and the differences in volume between the pipette and intracellular compartments were taken into account. This was necessary because the external adrenergic stimulus present in the new data represents a more physiological way of elevating cytosolic cAMP, which differs from the previous studied effects of “clamping” cAMP levels by the pipette. With this more precise representation of the pipette introduced, adrenergic signaling was included in the model by adding the β_3_ARs and their effect on intracellular cAMP (Fig. 2).

### The model can describe all previous data as well as new data involving external adrenergic stimulations

**Figure 3:**
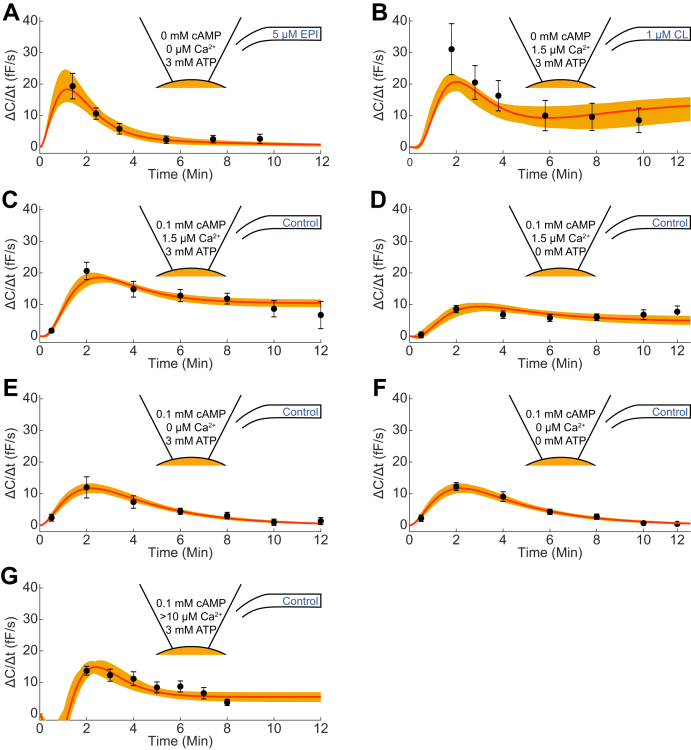
**The model performance on estimation data, consisting of both intracellular pipette and extracellular adrenergic stimulation.***A*–*G*, the *black dots* and error bars represent mean and SEM values of the experimental data (measured in femtofarad/second, fF/s), the *red solid line* represents the model simulation with the statistically best parameter set, and the *orange area* represents the model uncertainty. The exact IC and EC conditions are described within the symbolized pipette tip (different concentrations of cAMP, free Ca^2+^ and ATP) and within the symbol representing the glass capillary for the EC inlet (control, EPI or CL). The free [Ca^2+^] was calculated in (8). *A*, n = 4 to 6, (*B*) n = 6 to 7, (*C*) n = 8, (*D*) n = 7, (*E*) n = 9, (*F*) n = 13, (*G*) n = 9 to 10.

Figure 3 depicts the data used for developing the new model where each subfigure represents how a combination of extracellular (EC; adrenergic) and intracellular (IC; constituents of the pipette solution) input affects the cell. The new data including the response to external stimulation are shown in Figure 3, *A* and *B*, and the old data used in the development of the previous model (pipette stimulation only (9)) are shown in Figure 3, *C*–*G* (details are described in the legends). Adiponectin exocytosis is stimulated either by epinephrine (EPI) or CL with different concentrations of cAMP, ATP, and Ca^2+^ included in the pipette solution (IC). The experimental data points are denoted by black dots and error bars representing the mean and SEM values. The model simulations are shown as solid red lines for the best agreement with data (with optimal parameter set ${\text{θ}}_{\text{est}}^{\text{∗}}$, values given in Table 1), and the orange area represents the bounds of the model uncertainty. The model uncertainty was gathered by maximizing and minimizing the simulation in each experimentally measured time point, while still requiring a cost below the χ^2^-threshold. Clearly, the model agrees well with data, both for the EC and the IC stimulus data. This visual assessment of the good agreement is statistically supported by the fact that the model passes a χ^2^-test ($\cos\text{t}\left({\text{θ}}_{\text{est}}^{\text{∗}}\right)$ = 28.4 < χ^2^(0.05, 24) = 36.4; Experimental procedures). As can be seen, the model uncertainties are reasonable and limited. In summary, the new model can describe all previous IC data as well as new data involving both IC and EC stimulation.

**Table 1:** Optimal parameter values

Parameter	Value
Krel	3.7356 · 10^−4^
kexo	2.3764 · 10^−2^
kCaATP	6.5771 · 10^1^
kcAMP	5.9263 · 10^−2^
kCa2	2.4300 · 10^−4^
kATP2	2.2347 · 10^−2^
kEndo	5.8257 · 10^2^
kCacAMP	1.7826 · 10^1^
Km	7.2790 · 10^−3^
kdegcAMP	3.3182 · 10^−4^
kremCa	1.2482 · 10^−2^
kdegATP	1.9718 · 10^3^
kDiffcAMP	2.0835 · 10^−2^
kDiffCa	4.4218 · 10^−2^
kDiffATP	3.4721
kB	3.9240
kCL	5.6875 · 10^−3^
k1	2.2070 · 10^−3^
k2	2.4024 · 10^−2^
k3	1.0046 · 10^−4^
Vpip	2.1168 · 10^−5^
Vcell	1.3990 · 10^−13^

The optimal values found for the model parameters during parameter estimation. All parameters except Vpip and Vcell were given a free range between 10^−4^ and 10^4^ during the parameter estimation. Vpip was given a range between 2·10^−5^ and 6·10^−5^ (20–60 μl, estimated volume of the pipette). Vcell was given a range between 8.6·10^−15^ and 171.7·10^−15^ (8.6–171.7 fl, estimated volume of the cell).

### The model is able to describe independent validation data involving other combinations of stimuli not used for model development

**Figure 4:**
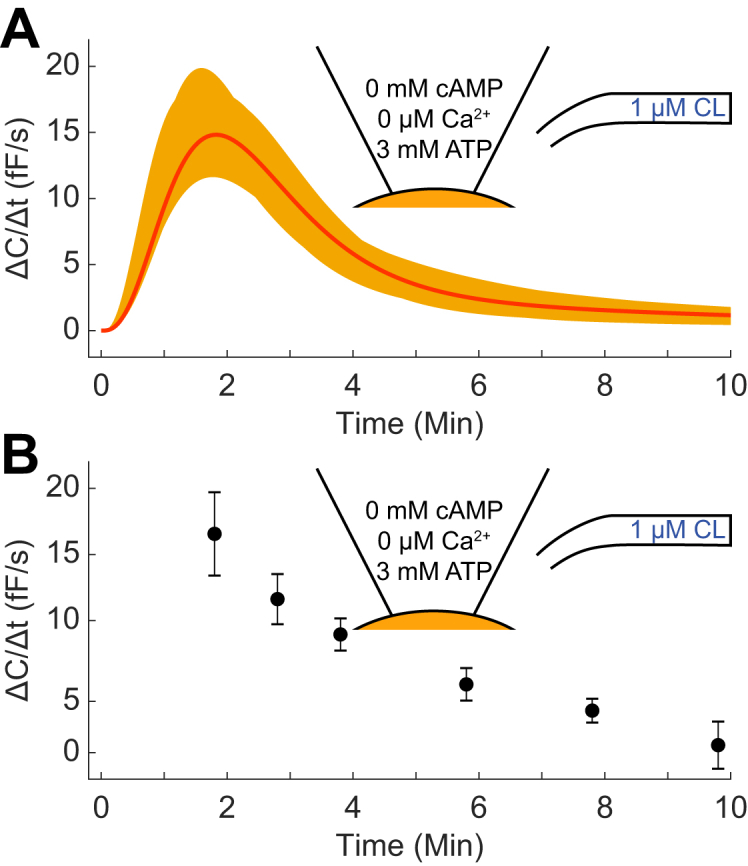
**Independent model prediction and corresponding experimental data.***A*, the stimulation with 1 μM CL EC in the presence of 3 mM ATP IC was predicted using the model. The *solid red line* represents the best parameter set with respect to the estimation data, and the *orange area* signifies the model prediction uncertainty. *B*, the same experiment was performed experimentally, and the rate of exocytosis was measured at the indicated timepoints (measured in femtofarad/second, fF/s). *Black dots* and error bars denote mean and SEM values of the experimental data (n = 6–8).

We next tested if the new model is able to correctly predict new independent validation data. The validation experiment consists of a combination of EC and IC inputs: 1 μM CL and 3 mM ATP, respectively (Fig. 4*A*). The prediction uncertainty was gathered in the same way as the model uncertainty, by maximizing and minimizing the prediction. Since the prediction has a low uncertainty (less than 30% at the peak value), it is meaningful to compare the prediction with experimental data, to test the quality of the model. This experimental data is shown in Figure 4*B*. As can be seen, both quantitative and qualitative aspects are in agreement. At t = 2, simulations show a peak value between 11 and 20 fF/s, which is in excellent agreement with the experimental range of 13 to 20 fF/s. Note that it is not important that the ranges are identical, but only that they are overlapping. The agreement is also statistically confirmed by the fact that the model passes a χ^2^-test, with the optimal parameter set from the estimation ($\cos\text{t}\left({\text{θ}}_{\text{est}}^{\text{∗}}\right)$ = 12.1 < χ^2^(0.05, 6) = 12.6).

### The model predicts a decline in peak secretion upon a decrease of β3 adrenergic receptors

**Figure 5:**
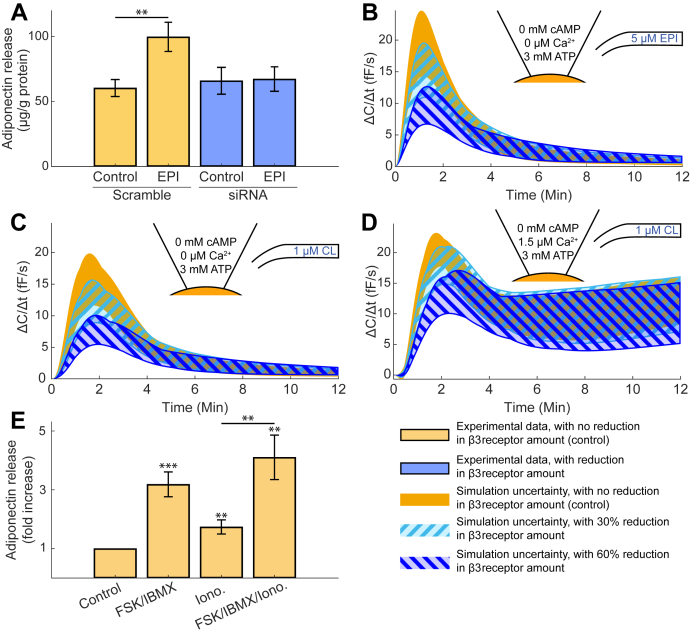
**Model prediction of a 30% and 60% decrease in β3 adrenergic receptors.***A*, experimental data (mean and SEM, n = 11) of absolute adiponectin secretion in control (scramble) and siRNA-treated 3T3-L1 adipocytes, incubated with epinephrine through 30 min. Note that this data is produced from the experimental series in Figure 7F of (5). *B*–*D*, model simulations upon downregulation of β_3_ARs (in femtofarad/second, fF/s). In all subfigures, the *orange area* corresponds to the prediction without reducing the amount of receptors, and the striped areas represents the prediction with a reduction of the amount of β_3_ARs by 30% (*cyan*) and 60% (*blue*). *E*, experimental data (mean and SEM, n = 8) of adiponectin secretion (fold over basal) in control (unstimulated) 3T3-L1 adipocytes or in adipocytes stimulated with either FSK/IBMX, Ionomycin (Iono.), or the combination of FSK/IBMX and Iono. ∗*p* < 0.05, ∗∗*p* < 0.01, ∗∗∗*p* < 0.001.

The validated model can be used to predict the outcome of new experiments in single cells, given the mechanisms included in the model. To illustrate this capability, we simulated a new experiment that would be challenging to carry out experimentally. The simulated *in silico* experiment was inspired by published data demonstrating that adrenergically triggered adiponectin secretion (adiponectin collected during a 30 min static incubation) was almost completely abrogated in primary adipocytes isolated from obese and diabetic mice. This blunted secretion was associated with a 30% lower abundance of β_3_ARs (8), a condition we can also include in the model. Figure 5*A* shows our own adiponectin secretion data from an experiment, where cultured 3T3-L1 adipocytes were genetically ablated for β_3_ARs (small interfering RNA reducing gene expression by ∼60%) and stimulated with epinephrine during a 30 min static incubation. As can be seen, adiponectin release is increased twofold in control (scramble) cells exposed to epinephrine, whereas siRNA-treated adipocytes show a nonsignificant response. To experimentally investigate how reduced expression of β_3_ARs affects the molecular regulation of adiponectin exocytosis by applying single cell patch-clamp to siRNA-treated cells is demanding. We therefore used our model to examine the corresponding predictions *in silico*. The amount of β_3_AR was downregulated by 30% or 60%, thus corresponding to the two experiments described above, and the resulting exocytosis response was simulated. Figure 5*B* shows the result, where the simulation consists of a combination of 5 μM EPI (EC) and 3 mM ATP (IC; no cAMP or Ca^2+^ included), for both control (orange area) and perturbed (blue striped areas) cells. As can be seen, the 30% and 60% downregulation of β_3_ARs is only translated into an approximate 20% and 47% decrease in peak exocytosis respectively; thus single-cell adiponectin exocytosis as understood by our model is significantly less affected than the experimental data on secretion indicates. As demonstrated in Figure 5*C*, simulation with 1 μM CL EC, under the same IC conditions as in 5B, yielded similar results. As shown in Figure 5*D*, inclusion of Ca^2+^ (IC) in the model partly rescues the reduced response. All ranges of peak exocytosis inhibitions are given in Table 2.

**Table 2:** Model prediction of peak exocytosis inhibition

Experimental conditions		Inhibition (%)	
		β_3_AR ↓30%	β_3_AR ↓60%
I	5 μM EPI, 3 mM ATP	20	47
II	1 μM CL, 3 mM ATP	21	48
III	1 μM CL, 3 mM ATP, 1.5 μM Ca^2+^	9	24

Summary of the predicted inhibition of adiponectin secretion upon a 30% and 60% reduction of β3ARs, using our new model for single cells. The three conditions I to III correspond to Figure 5, *B*–*D*, and the quantifications are done for the parameter set resulting in the largest prediction, at the time point of the highest secretion.

To test the effect of an intracellular Ca^2+^ elevation on adiponectin secretion in a population of cells, 3T3-L1 adipocytes were incubated (30 min) in the presence of the adenylyl cyclase activator Forskolin (FSK; 10 μM) and the phosphodiesterase inhibitor Isobutylmethylxanthine (IBMX; 200 μM), and/or with the Ca^2+^ ionophore ionomycin. FSK/IBMX elevates 3T3-L1 adipocyte intracellular cAMP to high levels (5), and ionomycin potently increases adipocyte intracellular Ca^2+^ (6). As expected, FSK/IBMX stimulated adiponectin release. In line with data in Figure 5*D*, adiponectin secretion was induced also by the ionophore ionomycin alone (although of a lower magnitude compared with FSK/IBMX) and the ionophore tended to increase adiponectin release stimulated by FSK/IBIX (Fig. 5*E*).

### The model can be used to investigate the intracellular dynamics

**Figure 6:**
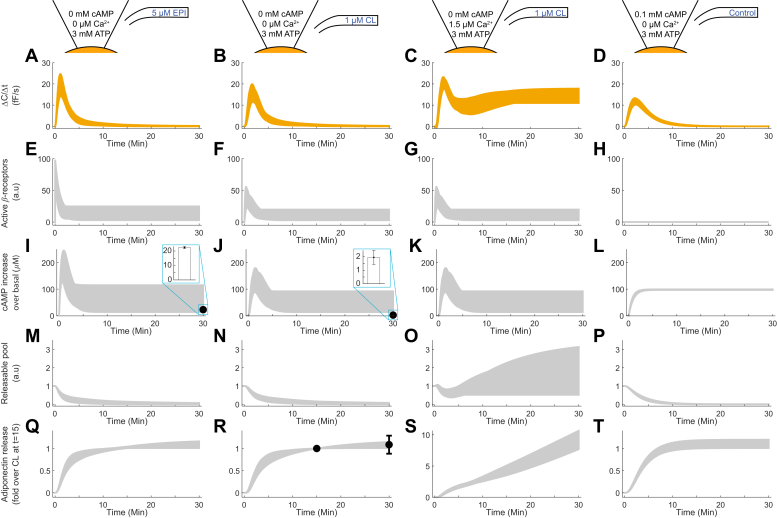
**Model simulations of a selection of states and experiments.** Each row corresponds to a different model state, as indicated in the *left-most* axis label: rate of change (*A*–*D*), active β-receptors (*E*–*H*), cAMP increase over basal levels (*I*–*L*), the releasable pool (*M*–*P*), and adiponectin secretion (*Q*–*T*). See Figure 2 for a graphical representation of the model. Columns correspond to experimental conditions described in the experimental summary at the *top row*: EPI without Ca^2+^ (*A*, *E*, *I*, *M* and *Q*), CL without (*B*, *F*, *J*, *N* and *R*) or with (*C*, *G*, *K*, *O* and *S*) Ca^2+^, and control with cAMP in the pipette (*D*, *H*, *L*, *P* and *T*). Mean and SEM of experimental data from batch experiments are represented as error bars (n = 5 in (*I*), n = 7 in (*J*), and n = 9–15 in (*R*)).

We used our validated model to investigate the intracellular dynamics. With the validated model we can observe all model variables under all considered experimental conditions, even variables corresponding to entities that are currently experimentally unmeasurable. A selection of the model states and experimental conditions are presented in Figure 6. By analyzing the model variables we could determine that:

(1) The exocytosis is delayed with respect to the receptor activation. For experiments with EPI or CL, this delay is observed upon comparing the model states for the exocytosis (Fig. 6, *A*–*C*) and the receptor activation (Fig. 6, *E*–*G*) at the time of the peak. Clearly, the exocytosis peaks at a later time point compared with the receptor activation. This is true for all conditions, even when taking the uncertainty of the predictions into account.
(2) The exocytosis is halted when the releasable vesicle pool is depleted (*cf.* the model states for the exocytosis in Fig. 6, *A*–*D* with the releasable pool in Fig. 6, *M*–*P*). When the releasable vesicle pool is depleted (approaches zero), the exocytosis ceases. Note that in the experiment with CL and Ca^2+^ (Fig. 6*O*), the releasable pool is not depleted, due to Ca^2+^-dependent replenishment of vesicles residing in the reserve pool. Thus, in the experiment with CL and Ca^2+^, exocytosis is sustained.
(3) cAMP is rate-determining with regard to the exocytosis (*cf.* the temporal dynamics for peak exocytosis in Fig. 6, *A*–*D* with the peak for cAMP in Fig. 6, *I*–*L* in the experiments with EPI or CL). In all three experimental series (Fig. 6, *A*–*C* and *I*–*K*), the peaks follow the same temporal pattern. In the experiment without extracellular stimuli and with cAMP and ATP (without Ca^2+^) included in the pipette solution (Fig. 6*D*), the exocytosis follows the cAMP increase initially (Fig. 6*L*) but later drops despite a sustained high cAMP level. We ascribe this to the depletion of the releasable vesicle pool (Fig. 6*P*), related to 2) above.

To test the model predictions, we compared cAMP predictions with experimental data from a population of cells, and we collected new experimental data for adiponectin secretion (Experimental procedures). The predicted ranges of cAMP levels at 30 min overlap with the experimentally measured values for EPI stimulation (Fig. 6*I*, predicted: 13.4–114.8, measured: 22.4 ± 0.7) and are slightly above the measured values for CL stimulation (Fig. 6*J*, predicted: 13.2–91.6, measured: 2.0 ± 0.5; (8)). For adiponectin secretion, the model simulations needed to be scaled from one single cell to a population of cells. This was done by expressing both experimental data and model simulations as fold over the amount of adiponectin secreted after 15 min of CL stimulation. As shown in Figure 6*R*, the model prediction at 30 min (1.0–1.15) agrees well with the experimental data for CL-triggered adiponectin release (1.1 ± 0.2).

## Discussion

The aim of this work was to develop a comprehensive mathematical model of adiponectin exocytosis that includes adrenergic stimulus, a chief signaling pathway involved in the physiological regulation of adiponectin secretion. To the best of our knowledge, no such model exists. The final model can explain a combination of experimental data, and it can predict independent validation data as well as be used to simulate nontrivial predictions (alterations and measurements that are difficult to perform experimentally). Below we will discuss the usefulness of our model, as well as some of its limitations.

Although our mathematical model in (9) improved our understanding of the molecular regulation of adiponectin exocytosis, it was restricted to studying the role of intracellular cAMP, Ca^2+^, and ATP (5, 6). Our own more recent research has shown that adiponectin exocytosis is triggered by catecholamine signaling *via* activation of β_3_ARs (8). In the current work, we have therefore developed a model that is able to describe the combination of internal and external (adrenergic) stimulus (Fig. 3), which thus brings us closer to a representative physiological model of adiponectin exocytosis. The model can describe both previous and new data (Fig. 3), and it can accurately predict independent validation data not used for estimating the parameter values (Fig. 4). This agreement with the data is supported by statistical tests showing that the data could have been generated by simulations with the model plus random noise.

We used the validated model to predict how adiponectin exocytosis would be affected if the abundance of β_3_ARs was reduced by 30%. This magnitude of decreased β_3_AR protein expression has been observed in primary subcutaneous adipocytes isolated from mice with obesity-induced diabetes (8). As shown in Figure 5, *B* and *C* (striped areas), the model yields a decrease in the exocytosis amplitude upon stimulation with either CL or EPI, when β_3_ARs are reduced. However, the rather slight decline in exocytosis amplitude stands in contrast to the complete abrogation of secretion observed in the “obese” adipocytes (8). Although the simulations and the data represent different conditions, *i.e.*, a precise perturbation of single cell exocytosis *versus* a more imprecise disease condition in a population of cells, the observed differences argue that additional obesity-associated molecular disruptions need to be included in the current model structure. One such mechanism could be the concomitant 30% reduction of Epac1 in the obese adipocytes; the cAMP-triggered adiponectin secretion occurs *via* activation of this signaling pathway (8). The obesity-associated reduced abundance of Epac1 likely contributes to the complete quenching of adiponectin secretion in the isolated cells. This is supported by that siRNA silencing of Epac1 in 3T3-L1 adipocytes abrogates EPI-stimulated adiponectin secretion (8). Moreover, we have recently demonstrated that gene expression of the exocytotic proteins VAMP (Vesicle-Associated Membrane Protein) 2 and 4 is reduced in visceral mouse adipocytes isolated from obese and diabetic mice (12). From this quick overview, it is apparent that several additional regulatory steps involved in the control of adiponectin exocytosis should be investigated further. Such future work, applying the types of predictions that we present here, will drive both further development of the mathematical model and of associated experiments. In this integrated way, the model can be advanced and tested, to objectively refine and test our understanding of the adiponectin release mechanisms. The finding that the model can explain experimental data using both EPI and CL supports our previous suggestion that catecholamine-triggered adiponectin exocytosis occurs *via* activation of β_3_ARs and that the stimulation does not involve β_1_ or β_2_ adrenergic receptors (8).

Diving further into our predictions and related experiments, Figure 5 shows that even downregulating the β_3_AR expression by as much as 60% (blue striped area in Fig. 5, *B* and *C*) does not abolish exocytosis to the extent shown for secretion in Figure 5*A*. This is perhaps more perplexing than the difference between model and experimental data upon 30% downregulation of β_3_ARs, since the data in Figure 5*A* represents an siRNA knockdown system, which can be regarded to be more “pure,” compared with a decreased expression owing to a pathophysiological condition. A plausible explanation for this second lack of agreement is that we compare responses in a population of cells during static conditions (secretion) with dynamic events in one single adipocyte (model). We cannot rule out the possibility that: (1) some adipocytes in Figure 5*A* in fact release adiponectin in response to stimulation but that this population is too small to result in significantly elevated amounts of adiponectin in the medium; (2) adipocytes in the static incubations release substances that act on the cells in an autocrine or paracrine fashion to affect further secretion. Another alternative explanation is that a certain threshold level of β_3_ARs is required to maintain the functionality of the β_3_AR-cAMP-adiponectin signaling. Both cAMP and Ca^2+^ signaling is known to be contained to functional microdomains where signaling proteins and regulatory factors can interact (13, 14). A 30% decreased abundance of β_3_ARs is perhaps enough to critically disturb this organization. It should moreover be emphasized that the 60% siRNA knockdown represents gene expression and that we do not have data representing β_3_ARs protein level. Again, all these mechanisms can and should be explored in future integrated experimental-modeling work.

As already described, Ca^2+^ alone is unable to stimulate exocytosis in 3T3-L1 adipocytes depleted of cAMP (lacking in the pipette solution) (5). In light of this, it may appear confusing that the elevation of intracellular Ca^2+^ in our model rescues the exocytosis response in adipocytes with reduced β_3_ARs (Fig. 5*D* and Table 2). However, the secretion data in Figure 5*E* demonstrates that adiponectin secretion can be induced by an elevation of intracellular Ca^2+^ in adipocytes where endogenous cAMP levels are maintained. We hypothesize that Ca^2+^, although unable to trigger exocytosis of adiponectin on its own, can act together with a nonstimulatory concentration of cAMP to induce secretion. It will be interesting to do joint experimental and modelling investigations to study this in more detail.

Using the model, we could find three biologically relevant insights (Fig. 6): (1) the exocytosis is delayed with respect to the receptor activation, (2) the exocytosis is halted when the releasable pool is depleted, and (3) cAMP is rate-determining with regard to the exocytosis. That the exocytosis is delayed with respect to the receptor activation is reasonable, since intracellular machinery, such as EPAC, and the production of cAMP, must be activated before the vesicles can fuse with the membrane. The finding that the exocytosis is halted upon depletion of the releasable vesicle pool in the absence of Ca^2+^ is in agreement with our own experimental observations (5) as well as with our previous modeling work (9). In all experiments, the temporal dynamics of cAMP closely resembles those of the exocytosis, as long as the releasable pool is sufficiently filled. This is reasonable since cAMP is involved in the final step of vesicle fusion with the plasma membrane. Clearly, if the releasable pool is devoid of vesicles, exocytosis cannot occur, and the temporal behavior of cAMP is not mimicked in this situation (see Fig. 6, *D* and *L*). Collectively, the predictions in Figure 6 are rational given what we know about the system; they demonstrate that the exocytotic mechanistic machinery that underlies the model’s simulations is consistent with biological knowledge and reasoning.

There are some technical details regarding the comparison with data that warrants further comments. For instance, some model simulations lie outside of the data uncertainty (*cf.* Fig. 3*B*, t = 2, and Fig. 3*G*, t = {6,8}), but the agreement is still statistically acceptable. The model is trained to 47 data points, and to have a statistically acceptable agreement between model simulation and data, not all these data need to be in perfect agreement. Looking at Figure 3*B*, the uncertainty in t = 2 is rather large and the cost of missing that data point is therefore small. In Figure 3*G*, on the other hand, the uncertainty is rather small, and the cost of deviating the same distance from these data points is therefore higher. Since the model is statistically acceptable, there is no strong argument to improve the agreement to either of these data points. Moreover, to attain better agreements with the estimation data in Figure 3, we would likely need to implement more detailed mechanisms. This could result in an overfitted model (*i.e.*, a model that includes variations that are not representative of the “true” model structure), a poorer agreement with validation data (Fig. 4), and/or a higher uncertainty of the new predictions (Figs. 4*A* and 5). In Figure 6, the model predictions for single cell conditions were compared with experimental data from a population of cells. Furthermore, the model predictions in Figure 6 were extrapolated to 30 min, which is beyond the 12-min window used for training the model (Fig. 3). While the relatively successful corroborations of the model with the experimental data provide some support for the model, such extrapolations to other time points and conditions go toward the boundary of what a model can be expected to accurately predict.

Another technical comment concerns the way we calculated these model uncertainties. The uncertainties were obtained by solving a modified optimization problem, suggested in (15), for each condition (minimization/maximization, experiment, and time point). The reformulated problem yielded wider estimations of the model uncertainties, compared with simply restarting the search for optimal parameter sets multiple times and saving all parameter sets (results not shown). In both these approaches, only parameter sets with sufficiently good agreement to data were used. We did not compare the reformulated problem against Markov Chain Monte-Carlo sampling (16) or prediction profile likelihood (17), but under the assumption that the reformulated problem is solved to optimality, our approach should yield at least an equally strong estimation of the uncertainties. Another aspect is that the uncertainties of the model simulations have different widths under the various experimental conditions; compare *e.g.*, Figure 3, *B*–*D*. These different widths are explained by that the model uncertainty is largest in the datasets with the largest measurement uncertainty. Finally, this approach implies that we do not have uniquely identifiable parameter values, which means that the optimal parameters in Table 1 are not unique. Therefore, the parameter values might not be directly comparable to values of other models.

In conclusion, we here present the first model able to explain effects of both intracellular and extracellular cues on adiponectin exocytosis. The model is clearly useful for prediction of experimental data and shows promises to be valuable for gaining future mechanistic and molecular insight that would be difficult to obtain experimentally. In the more long-term perspective, these model-based insights can also be useful for medical applications. Obesity-associated diabetes is a common and costly disease, with severe complications, such as cardiovascular dysfunction, blindness, and kidney failure (18). In order to facilitate both prevention and treatment of diabetes, it is important to understand and study the mechanisms involved in metabolic regulation, as well as aberrations associated with disease development. Hypoadiponectinemia is one of several hallmarks in obesity-associated diabetes (4). We think that the work presented here has promising prospect to help solving a part of the puzzle, by enabling us to gain new knowledge about the pathophysiological regulation of adiponectin exocytosis. However, the progression into type 2 diabetes is the result of the breakdown of a complex interplay between several different organs, hormones, metabolites, and other factors. This complex interplay consists of several interacting subsystems, and both these subsystems and their interactions need to be understood, in order to fully comprehend the disease. The current model of adiponectin exocytosis cannot explain how adiponectin is controlled on an organ or whole-body level. An exciting way forward is to bring our model into the larger picture of the whole-body energy homeostasis, to both increase our understanding of normal physiology, and to acquire new knowledge of how adipose tissue dysfunction is connected to the development of type 2 diabetes and its associated comorbidities. Such advances can be done by combining our model with existing models at the molecular, organ, and whole-body level (19, 20, 21).

## Experimental procedures

### Mathematical modeling

#### Model formulation

The model was constructed using ordinary differential equations (ODEs) in MATLAB and solved numerically using IQM tools, a continuation of SBtoolbox (22) available at https://iqmtools.intiquan.com, with Sundials CVODEs (23). All interactions and regulations in the model are illustrated in Figure 2. Below we present the model equations in detail. For simplicity and readability, we will sometimes group and go through equations together, as well as write out some rates explicitly on the right-hand side of the ODEs that are otherwise defined as intermediate variables in the model files in the code repository (see Data availability).

The most central component of this extended model is the β_3_AR. Simplified, this receptor oscillates between three conformational states: one active, one inactive state as well as a desensitized (internalised) state. The receptor is activated by adrenergic stimuli, such as epinephrine. Post activation, the receptor will be desensitized and becomes unavailable for further stimulus until it has again been resensitized and returned to the inactivated state. We model the β_3_AR using the ODEs in Equation 1

(1) $$\begin{array}{l} d/dt\left(B\right)\;=\;k1\cdot Bde\;-\;kB\cdot B\cdot \left(EPI\;+\;kCL\cdot CL\right)\\ d/dt\left(Bact\right)\;=\;kB\cdot B\cdot \left(EPI\;+\;kCL\cdot CL\right)\;-\;Bact\cdot k2\\ d/dt\left(Bde\right)=\;Bact\cdot k2\;-\;k1\cdot Bde \end{array}$$

where *B* and *Bact* represent the inactive and active states, respectively; where *Bde* is the state of desensitization, in which the β_3_AR is unavailable; where the activation of the inactive receptor *B* occurs in response to either epinephrine (*EPI*) or *CL*; where *kCL* is a parameter that offsets the level of activation when stimulating with CL, to account for the difference in activation strength between EPI and CL; where *kB*, *k1*, and *k2* are kinetic rate parameters, determining the level of activation due to adrenergic stimuli, passive resensitization of the desensitized receptor, and desensitization of the activated receptor, respectively.

When the β_3_AR has been activated, it will trigger an intracellular production of cAMP, *via* activation of adenylyl cyclase. Unfortunately, we do not have experimental data for the activation of adenylyl cyclases. Therefore, we simplify the full process as a direct action from β_3_AR on the endogenous production of cAMP. External cAMP can also be infused *via* the patch pipette and cAMP can be degraded. We have modeled intracellular cAMP and cAMP in the pipette in Equation 2

(2) $$\begin{array}{l} d/dt\left(cAMP\right)\;=\;kDiffcAMP\cdot \left(pipcAMP-cAMP\right)\cdot pip\;+Bact\cdot k3-kdegcAMP\cdot cAMP\;\\ d/dt\left(pipcAMP\right)\;=\;-\;kDiffcAMP\cdot \left(pipcAMP\;-\;cAMP\right)\cdot pip\cdot Vcell/Vpip\; \end{array}$$

where the production of cAMP depends on the level of activation of the β_3_AR (*Bact*), and the parameter *k3*; where the degradation of cAMP depends on the concentration of cAMP, and the parameter *kdegcAMP*; where the levels of cAMP can be changed by attaching the pipette; where the presence or absence of the pipette is determined by the binary parameter *pip*. Note that the difference between the pipette concentration of cAMP (*pipcAMP*) and the intracellular concentration of cAMP (*cAMP*) determines the direction in which cAMP will diffuse and that the diffusion rate is determined by the parameter *kDiffcAMP*. Diffusion can only occur if the pipette is firmly attached to the cell. To account for the large difference in volume between the cell (*Vcell*) and the pipette (*Vpip*), the quotient of the volumes was used to scale the diffusion to the pipette.

Beyond cAMP, the intracellular mediators Ca^2+^ and ATP were also used; in both cases the stimulation occurs *via* diffusion from the patch pipette. The equations for Ca^2+^ and ATP are essentially the same as for cAMP, except for the absence of an intracellular production. The ODEs are given in Equation 3

(3) $$\begin{array}{l} d/dt\left(Ca\right)\;=\;kDiffCa\cdot \left(pipCa\;-\;Ca\right)\cdot pip\;-\;kremCa\cdot Ca\\ d/dt\left(ATP\right)=kDiffATP\cdot \left(pipATP-ATP\right)\cdot pip-kdegATP\cdot ATP\\ d/dt\left(pipCa\right)\;=\;-\;kDiffCa\cdot \left(pipCa\;-\;Ca\right)\cdot pip\cdot Vcell/Vpip\\ d/dt\left(pipATP\right)\;=\;-\;kDiffATP\cdot \left(pipATP\;-\;ATP\right)\cdot pip\cdot Vcell/Vpip \end{array}$$

where *Ca* and *ATP* represent the intracellular concentrations of Ca^2+^ and ATP; where *pipCa* and *pipATP* represent the concentrations of Ca^2+^ and ATP in the pipette; where *kDiffCa* and *kDiffATP* are parameters determining the rate of diffusion; and where *kremCa* and *kdegATP* are parameters determining the rate of removal and degradation, respectively.

The intracellular mediators cAMP, Ca^2+^, and ATP determine the rates by which stored vesicles, such as vesicles containing adiponectin, are exocytosed. Furthermore, these mediators also affect the rate of endocytosis (plasma membrane retrieval). The vesicle to be exocytosed will transit through different functional stages or pools. Firstly, a large pool of vesicles will be available as a reserve pool. Over shorter timescales, such as the rapid exocytosis modeled in this project, this pool will essentially not change in size and is therefore considered to be constant. Vesicles destined to undergo exocytosis will go through several maturation steps and thereafter belong to a pool we refer to as the releasable pool. Both Ca^2+^ and ATP are mediators that affect those maturation steps. Furthermore, the effect of ATP on vesicle maturation also depends on Ca^2+^. Vesicles in the releasable pool can, in response to a triggering signal (cAMP), fuse with the plasma membrane and undergo exocytosis (augmented by Ca^2+^ and ATP). Alternatively, the vesicles can transit back to the releasable pool. Both the exocytosis and the transition back to the releasable pool are passive effects, unaffected by cAMP, ATP, and Ca^2+^. The ODEs for these steps of exocytosis are given in Equation 4

(4) $$\begin{array}{l} d/dt\left(Res\right)\;=\;0\\ d/dt\left(Rel\right)=\;vRes\_Rel\;-\;vRel\_PM\;+\;krel\cdot PM\\ d/dt\left(PM\right)=\;vRel\_PM\;-\;krel\cdot PM\;–\;kexo\cdot PM\\ vRes\_Rel=\;\left(\left(Ca/\left(km+Ca\right)\right)\cdot \left(kCa2+kATP2\cdot ATP\right)\right)\cdot Res\\ vRel\_PM=\;cAMP\cdot \left(kcAMP\;+\;\left(Ca/\left(km+Ca\right)\right)\cdot ATP\cdot kCaATP\right)\cdot Rel \end{array}$$

where *Res*, *Rel*, and *PM* are model states for the different pools of vesicles in the cell, respectively representing the endless reserve, the pool of releasable vesicles, and the pool of vesicles that are fused with the plasma membrane; where the transport between the reserve pool and the releasable pool is given by *vRes_Rel*; where *km* is the parameter for the Michaelis–Menten constant of the saturating expression of *Ca* on the transport; where *kCa2* is parameter for the maximal response of the ATP-independent effect of Ca^2+^; and where *kATP2* is the parameter for the maximal response of the Ca^2+^ augmented ATP-dependent effect on the transport. The fusion of vesicles from the releasable pool with the plasma membrane is given by *vRel_PM*. Fusion triggered by cAMP alone is determined by the parameter *kcAMP* and the augmentation by Ca^2+^ and ATP is determined by the parameter *kCaATP*. Note that cAMP is required for any fusion with the plasma membrane to occur. The vesicles that are fused with the plasma membrane (*PM*) can either undergo exocytosis or be transported back to the releasable pool (*Rel*). These rates are determined by the parameters *kexo* and *krel*, respectively.

Upon exocytosis, the size of the plasma membrane will increase. To circumvent excessive and detrimental membrane area enlargement, exocytosis is followed by endocytosis. Endocytosis likewise depends on the concentrations of Ca^2+^ and cAMP (note that cAMP is required to trigger exocytosis prior to endocytosis), and the rate is determined by the parameter *kCacAMP*. The ODE for the endocytosis is given by Equation 5

(5) d/dt(Endo)=kCacAMP·cAMP·Ca−kEndo·Endo

where *Endo* represents a portion of the membrane, that is ready to undergo vesicular endocytosis, and where the rate of vesicle endocytosis is determined by the parameter *kEndo*.

The exocytosed vesicles will release adiponectin to the surrounding environment, where adiponectin will subsequently diffuse away from the cell. Due to the large volume of the extracellular environment in our experiments, it is unlikely that adiponectin will remain in close vicinity to the cells. Thus, uptake of adiponectin back into the cell during endocytosis is negligible. Therefore, we modeled adiponectin release to be proportional to the exocytosis. The ODE for the released adiponectin is given by Equation 6

(6) d/dt(Adiponectin)=kexo·PM

Where, just as in Equation 4, *PM* is the state for the pool of vesicles that are fused with the plasma membrane, and *kexo* is the rate-determining parameter for the exocytosis.

In the experimental data, the rate of change of the capacitance over the plasma membrane has been measured, as a proxy for the change in cellular size. To get a valid representation of the capacitance change, we set up a measurement equation in the model equal to the difference in exocytosis and endocytosis, scaled with an experimental measurement constant (Equation 7)

(7) $$\hat{y}=\;kscale\cdot \left(vExo-vEndo\right)\;=\;kscale\cdot \left(PM\cdot kexo-kEndo\cdot Endo\right)$$

where *kscale* is the measurement scaling constant. In practice, this constant was not part of the equation in the model file and was determined using MATLAB’s LSCOV function.

The full model equations are given in the GitHub/GitLab repositories (see Data availability below) in the file *model_equations.txt*.

Since the exact values of the rate-determining parameters (*krel*, *kexo*, *kCaATP*, *kbasal*, *kCa2*, *kATP2*, *kEndo*, *kCacAMP*, *km*, *kdegcAMP*, *kdegCa*, *kdegATP*, *kdiffPipcAMP*, *kdiffPipCa*, *kdiffPipATP*, *kB*, *kCL*, *k1*, *k2*, and *k3*) and the volumes of the cell and pipette (*Vpip* and *Vcell*) were unknown, these values had to be estimated based on the experimental data.

### Parameter and prediction uncertainty estimation

The performance of the model was evaluated using the objective function defined in Equation 8, commonly referred to as the cost of the model

(8) $$\text{cost}=\underset{\text{t}\in \text{T}}{\sum }\underset{\text{e}\;\in \;\text{E}}{\sum }{\left(\frac{{\text{y}}_{\text{t},\text{e}}-{\hat{\text{y}}}_{t,e}\left(\text{θ}\right)}{{\text{SEM}}_{\text{t},\text{e}}}\right)}^{2}$$

where y_t,e_ and SEM_t,e_ are the mean value and standard error of the mean (SEM) of the experimental data *e* at time *t*, T is the set of time points and E is the set of experiments, and ${\hat{\text{y}}}_{t,e}\left(\text{θ}\right)$ is the model simulation of experiment *e* at time *t*, depending on a set of parameters *θ*.

In order to evaluate the developed model, we use a *χ*^*2*^-test, which tests the null hypothesis that the experimental data have been generated by the model. In practice, the optimal cost given the optimal parameter set *θ∗* is compared against the *χ*^*2*^ test statistic, with *p* = 0.05 and the degrees of freedom equal to the number of datapoints (minus 1 for the scaling parameter introduced in the new model). If the cost is larger than the *χ*^*2*^-limit, the model is rejected.

Due to the sparsity of the data, and prior knowledge about when the peak exocytosis should occur in the extracellularly stimulated experiments, we added constraints and rejected parameter sets that did not fulfill these conditions.

The optimal parameter set (*θ*∗) was found by using a combination of MathWorks’ particle swarm (PS), simulated annealing (SA), and fmincon solvers. PS was used to generate starting points, SA to roughly tune the parameters, and fmincon to fine-tune the parameters. Firstly, multiple instances of a chain of PS-SA-fmincon were run in parallel to generate acceptable solutions. Secondly, a combination SA-fmincon was used to fine-tune the parameter values. In this second step, the previously best parameter set was used as the initial point for SA and the chain was restarted multiple times. In both situations, the parameter estimation schemes were run in parallel at the Swedish national supercomputing center (NSC).

The model uncertainty was estimated by maximizing and minimizing the model simulation in all available data points (*i.e.*, for all time points and all experiments) independently. To constrain the uncertainty, the cost was required to be below the optimal cost plus the *χ*^*2*^-statistic with *p* = 0.05 and one degree of freedom. A description of the optimization problem is given in Equation 9.

(9) $$\begin{array}{l} \min\;v={\hat{y}}_{e,t}\left(\theta \right)\\ s.t.\;cost\left(\theta \right)\;\leq \;limit\\ \mathrm{limit}\;=\;cost\left(\theta^{*}\right)+\chi^{2}\left(0.05,1\right) \end{array}$$

where ${\hat{y}}_{e,t}\left(\theta \right)$ is the model simulation of experiment *e* and time point *t*, depending on the parameters *θ*. For finding the maximum simulation, the objective function of Equation 9 was multiplied with −1 and solved as a minimization problem. In practice, the constraint (cost(θ)≤limit) was relaxed into the objective function as a penalty term. The reformulated optimization problem is given in Equation 10, where the penalty is only applied if cost(θ)>limit.

(10) $$\begin{array}{l} min\;v={\hat{y}}_{e,t}\left(\theta \right)+penalty\\ penalty=\left\{ \begin{array}{l} \left|{\hat{y}}_{e,t}\left(\theta \right)\right|+cost\left(\theta \right)\;-\;limit,\;when\;cost\left(\theta \right)>limit\\ 0\;else \end{array}\right. \end{array}$$

All subproblems were solved in parallel at NSC by using the current best estimation and running a chain of SA-fmincon, repeated multiple times.

### Experimental methods

Patch-clamp measurements of exocytosis and adiponectin secretion experiments are described in detail in (9). In short, 3T3-L1 cells were grown in plastic petri dishes (for patch-clamp recordings; Nunc) or 12-well plates (for secretion measurements; Sarstedt), and differentiation was induced by addition of a differentiation cocktail (5). For electrophysiological recordings, the adipocytes were superfused with an extracellular solution (EC) containing (in mM): 140 NaCl, 3.6 KCl, 2 NaHCO3, 0.5 NaH2PO4, 0.5 MgSO4, 5 Hepes (pH 7.4 with NaOH), 2.6 CaCl2 supplemented with 5 mM glucose. The pipette-filling solutions contained (in mM): 125 potassium glutamate, 10 KCl, 10 NaCl, 1 MgCl2, 5 Hepes (pH 7.15 with KOH) and were supplemented with cAMP, ATP, and Ca^2+^ as indicated. Cells were voltage clamped at −70 mV, and exocytosis was measured as increase in membrane capacitance in the standard whole-cell configuration, using an EPC-9 patch-clamp amplifier (HEKA Electronics) and PatchMaster software. Exocytosis rates (ΔC/Δt) were measured by application of linear fits (OriginPro: OriginLab Corporation) at indicated time points. Secreted adiponectin was measured in EC collected from 3T3-L1 adipocytes incubated with indicated secretagogues during 30 min (on gentle shaking) at 32 °C. At the end of the incubation, the EC was removed and centrifuged (2000 rpm, 5 min). Samples were aliquoted and stored at −80 °C. Secreted adiponectin (measured with mouse ELISA DuoSets; R&D Systems) was analyzed in relation to total protein content (Bradford protein assay). Small interfering RNA experiments were performed as described in (8).

## Data availability

All scripts and data used in this work are provided at https://github.com/willov/adiponectin-epi (https://doi.org/10.5281/zenodo.5226360) and are mirrored at https://gitlab.liu.se/ISBgroup/projects/adiponectin-epi.

## Conflict of interest

The authors declare that they have no conflicts of interest with the contents of this article.
